# P-1709. Utility of Catheter Tip Culture and Impact on Antimicrobial Therapy in Patients with Cancer

**DOI:** 10.1093/ofid/ofaf695.1881

**Published:** 2026-01-11

**Authors:** Wonhee So, Thu Phu, Jana Dickter, Suwannee Srisatidnarakul, Sanjeet S Dadwal, Rosemary She

**Affiliations:** Western University of Health Sciences, duarte, CA; Western University of Health Sciences, duarte, CA; City of Hope National Medical Center, Duarte, California; City of Hope, Duarte, California; City of Hope National Medical Center, Duarte, California; City of Hope, Duarte, California

## Abstract

**Background:**

Routine catheter tip culture (CTC) of central venous catheters (CVC) for diagnosing bloodstream infection (BSI) is not recommended per IDSA microbiology laboratory guidance. We evaluated if this practice impacts management of BSI as approximately 20% of CVC removal led to CTCs at our center.

**Figure:**
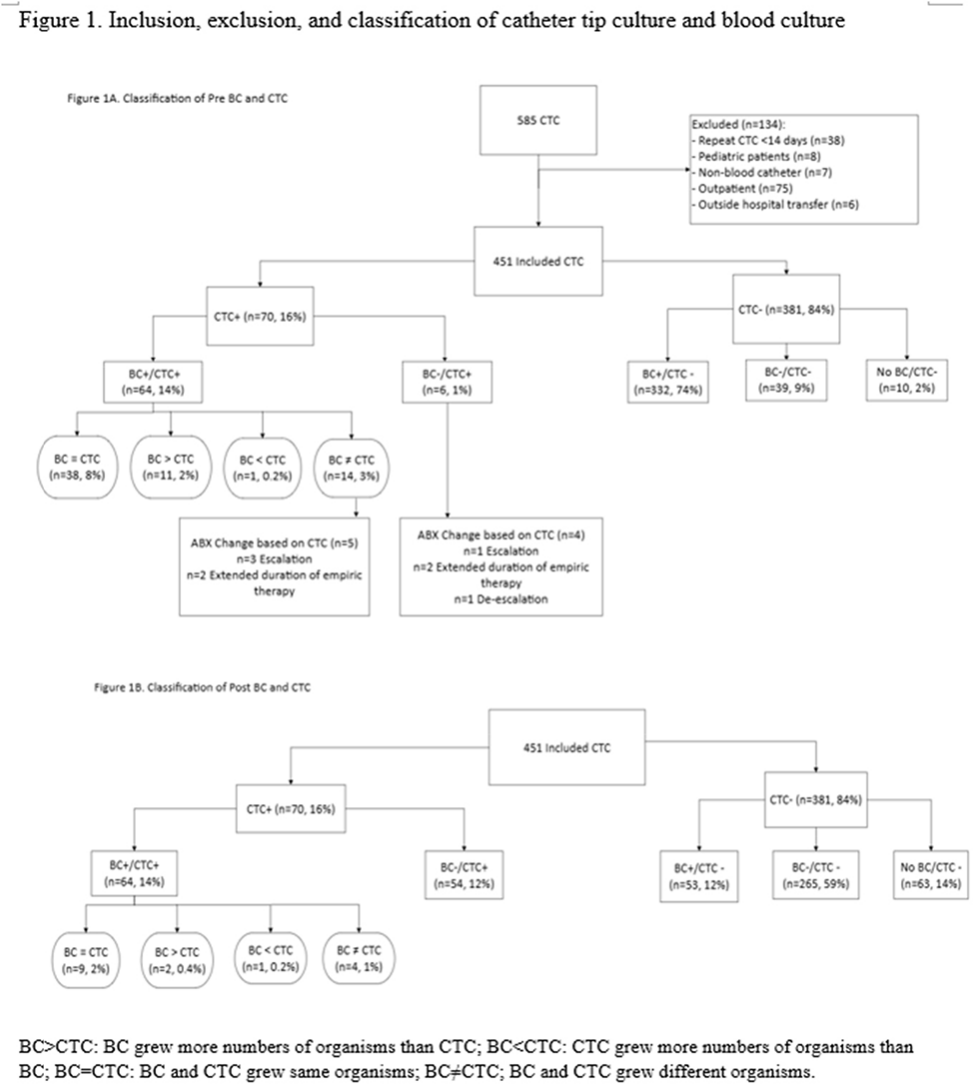

**Figure:**
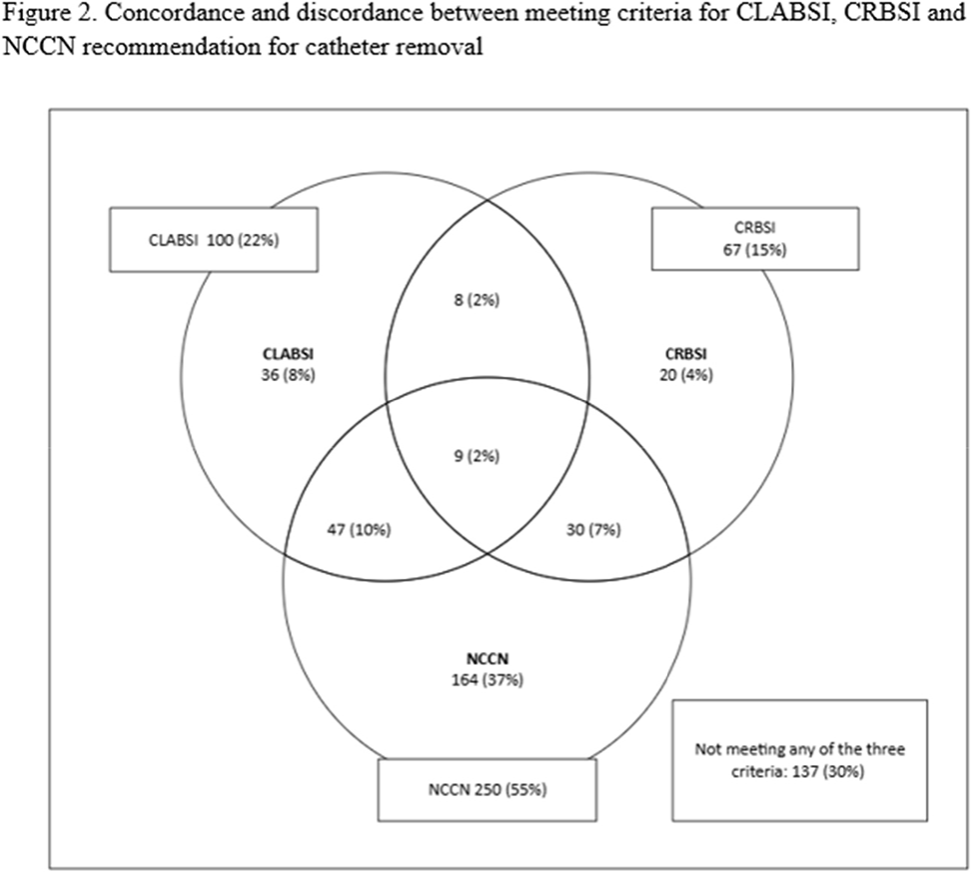

**Methods:**

Adult, hospitalized patients with CVC CTC performed between 2023-2024 were included in this retrospective, cross-sectional study at a comprehensive cancer center. Exclusion criteria were repeat CTC < 14 days, outpatients, and outside-hospital transfers. Patient characteristics, reasons for CVC removal, criteria for CVC removal per central line-associated bloodstream infection (CLABSI), catheter-related BSI (CRBSI) definitions, and National Comprehensive Cancer Network (NCCN) recommendations (Table 1), comparison between CTC and blood cultures 14 days pre- (preBC) and post- CTC (postBC), antimicrobial change based on CTC, and subsequent BSI data were collected. Descriptive statistics were performed using SPSS v.29.

**Figure:**
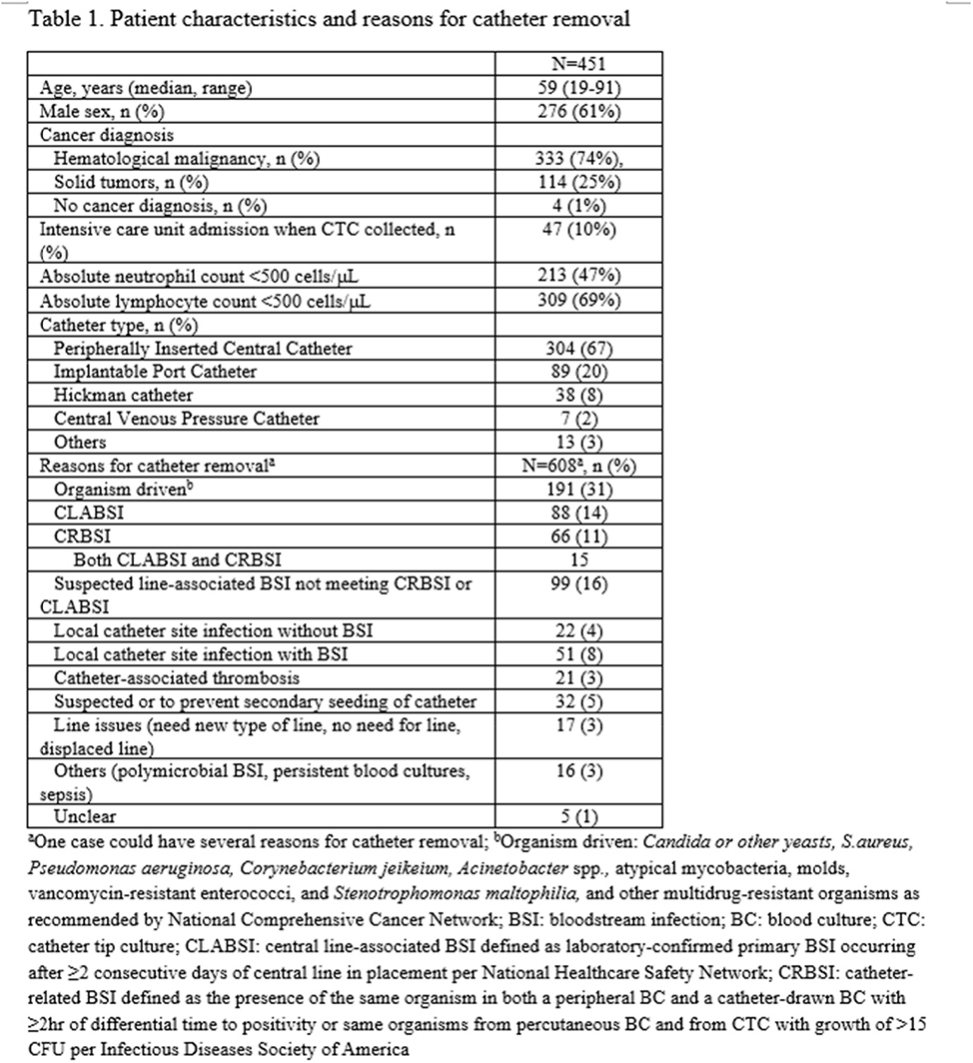

**Figure:**
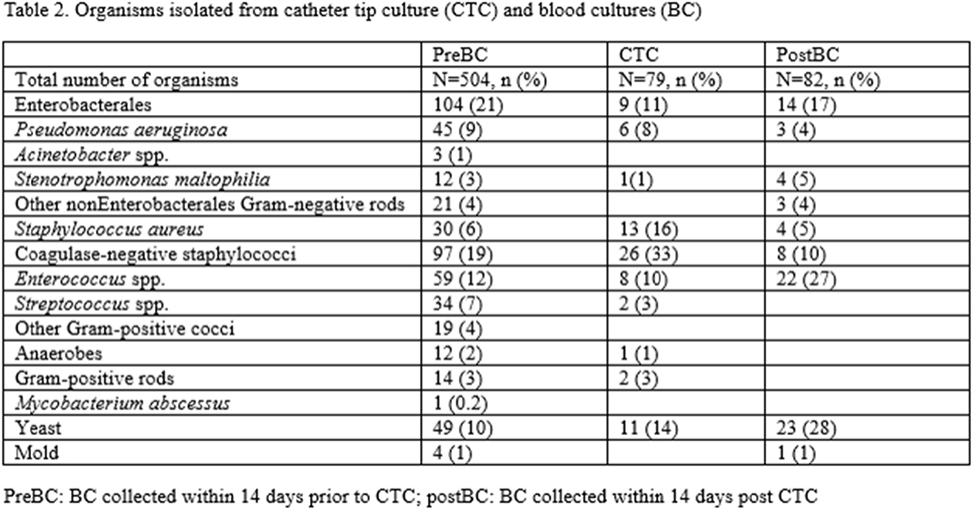

**Results:**

Eligible patients with 451 CTCs: median age 59 years, 61% male, hematologic malignancy 74%, and peripherally inserted central catheter 67% (Table 1, Figure 1A). Most common reasons for CVC removal were organism driven as recommended by NCCN (31%) and suspected line-associated BSI not meeting CRBSI or CLABSI (16%) (Table 1). Rates of meeting criteria for CVC removal per CLABSI (22%), CRBSI (15%), and NCCN recommendation (55%) varied with 30% not meeting any of the criteria (Figure 2). CTC was positive in 16% (70/451) with 79 organisms. Coagulase-negative staphylococci (33%) were most common among CTCs while Enterobacterales (21%) in preBCs and yeast (28%) in postBCs were most frequent (Table 2). 8% of CTC were concordant with preBC and 2% (9/451) discordant result led to antimicrobial changes (Figure 1A): 4 escalations, 4 with extended duration, and 1 de-escalation. 2% of CTC were concordant with postBC (Figure 1B).

**Conclusion:**

CVC removal in our practice was more aligned with NCCN recommendations. The low rates of concordant results for predicting CVC-associated BSI between CRBSI and CLABSI suggest lack of diagnostic utility of CTCs and minimal impact on management in this population, therefore should be avoided.

**Disclosures:**

Sanjeet S. Dadwal, MD, Ansun Biopharma: Grant/Research Support|Aseptiscope, Inc.: Stocks/Bonds (Private Company)|Basilea: Advisor/Consultant|Basilea: Grant/Research Support|F2G: Grant/Research Support|Karius: Advisor/Consultant|Karius: Grant/Research Support|Karius: Honoraria|Merck: Advisor/Consultant|Pfizer: Grant/Research Support|Pulmotect: Grant/Research Support|Symbio: Grant/Research Support|Takeda: Advisor/Consultant

